# Anterior Scleral Thickness in Young Saudi Adults Measured Using Swept-Source Optical Coherence Tomography: Sex- and Quadrant-Specific Variations

**DOI:** 10.3390/jcm15207847

**Published:** 2026-10-12

**Authors:** Naveen Kumar Challa, Nawaf M. Almutairi, Bandar M. Alenezi, Abdelaziz Mohamed Elmadina

**Affiliations:** Department of Optometry, College of Applied Medical Sciences, Qassim University, Buraidah 51452, Saudi Arabia; nm.almutari@qu.edu.sa (N.M.A.); b.alenezi@qu.edu.sa (B.M.A.); a.alkhalil@qu.edu.sa (A.M.E.)

**Keywords:** anterior scleral thickness, optical coherence tomography, swept source, Saudi Arabia, young adult

## Abstract

**Purpose:** To report baseline anterior scleral thickness (AST) values and evaluate regional and sex-related variation in healthy young Saudi adults using swept-source optical coherence tomography (SS-OCT). **Methods:** A prospective, cross-sectional study included 55 healthy Saudi adults aged 18–40 years. Participants underwent comprehensive ocular examinations to exclude pathology or significant refractive errors. AST was measured using IMAGEnet 6 software in superior, inferior, nasal, and temporal quadrants at 0 mm, 1 mm, and 2 mm from the scleral spur using the anterior segment module of swept-source OCT. Regional and sex-related differences were evaluated using a linear mixed-effects model adjusted for age and spherical equivalent. Short-term within-session intra-observer test–retest repeatability across repeated B-scan acquisitions was assessed using intraclass correlation coefficients, within-subject coefficients of variation, and Bland–Altman analysis. **Results:** The mean age of participants was 23.56 ± 4.61 years (34 males, 21 females). AST varied significantly according to distance and anatomical quadrant (both *p* < 0.001), with significant distance-by-quadrant (*p* < 0.001) and sex-by-distance-by-quadrant interactions (*p* = 0.010). The inferior quadrant generally showed greater thickness, while the superior quadrant showed lower values. Bonferroni-adjusted location-specific comparisons showed greater AST in males in the superior and temporal quadrants at both 1 mm and 2 mm from the scleral spur, whereas the remaining sex comparisons were not statistically significant. Neither age nor spherical equivalent showed a significant independent association with AST. Intra-observer ICC values ranged from 0.872 to 0.939. **Conclusions**: AST in young Saudi adults varies according to anatomical quadrant and distance from the scleral spur, with sex-related differences showing location-dependent patterns. These findings provide descriptive baseline data for anterior scleral morphology in this population.

## 1. Introduction

The sclera, a tough fibrous tissue, forms the outer wall of the eye and plays a vital role in maintaining the globe’s shape and mechanical stability. Beyond providing structural integrity, it serves as the attachment point for extraocular muscles, supports ocular biomechanics, and contributes to accommodation [1,2,3]. The anterior portion of the sclera also has a unique functional importance: by anchoring the scleral spur, it helps maintain aqueous humor outflow and intraocular pressure homeostasis [4,5,6]. Alterations in anterior scleral thickness (AST) have been linked to several ocular disorders, including scleritis, episcleritis, myopia, and glaucoma, that can cause inflammation, degeneration, or remodeling, which eventually change scleral properties [7,8,9,10,11,12,13].

Importantly, AST is not uniform across the eye. It varies by anatomical location and is influenced by biological and demographic factors such as age, sex, and refractive status. Advances in imaging techniques, particularly enhanced depth imaging (EDI) and swept-source optical coherence tomography (SS-OCT), have enabled more precise measurement of scleral thickness, showing that males and older individuals tend to have thicker sclera [14,15,16,17,18]. Previous studies have demonstrated substantial variation in AST between scleral meridians, with additional differences reported between population groups [7,15]. These observations are relevant when interpreting AST because measurements obtained from one quadrant, measurement distance, or population may not necessarily represent the anterior sclera as a whole. Furthermore, differences in imaging methodology, anatomical reference points, and participant characteristics may contribute to variation among published studies. Despite the increasing literature on anterior scleral morphology, data from Saudi adults remain limited, and the regional distribution of AST and its relationship with sex have not been well characterized. Therefore, the present study aimed to characterize baseline AST in healthy young Saudi adults using a standardized SS-OCT protocol and to evaluate its variation according to anatomical quadrant and distance from the scleral spur, while examining sex-related differences and accounting for age and refractive status. Establishing these baseline anatomical patterns may provide a useful framework for future studies investigating physiological variation and disease-related changes in the anterior sclera.

## 2. Methods

This cross-sectional study included 55 eyes from 55 eligible participants recruited from university optometry clinics in the central region of Saudi Arabia.

The study was approved by the university health research ethics committee (approval date: 6 January 2026; approval number 26-06-01) and was conducted in accordance with the tenets of the Declaration of Helsinki. Participant and data recruitment were conducted from 9th January 2026 to 15th May 2026. Of the 60 participants initially recruited, five subjects were excluded: two subjects because of poor cooperation during imaging, one subject because of suboptimal OCT image quality, and two subjects because ocular abnormalities (one with suspicion of glaucoma and the other with pinguecula) were identified during the clinical examination. Consequently, 55 participants were included in the final analysis. Among the included participants, 31 were recruited from outpatient clinics and 24 were healthy volunteers, including university students, staff members, and their family members.

The sample size was calculated for estimation of mean anterior scleral thickness. Based on a previously reported standard deviation of approximately 46 µm [16], a two-sided 95% confidence level (α = 0.05), and a desired precision of approximately ±15 µm around the estimated mean, a minimum sample of 37 participants was required using the formula *n* = (Zα/2σ/d)^2^. To improve precision and allow for exclusions due to image quality or ocular findings, 60 participants were initially recruited, of whom 55 were included in the final analysis. Because the original calculation was precision-based rather than hypothesis-test-based, statistical power and β were not applicable to this calculation.

Participants were recruited from university optometry clinics and among healthy volunteers, including students, university staff, and their family members originating from different regions of Saudi Arabia. Saudi nationality was self-reported at recruitment and verified using the participant’s Saudi national identification information entered during imaging registration; ethnicity was not independently assessed. Written informed consent was obtained from all participants before enrollment. Each participant underwent a comprehensive ocular examination, including visual acuity assessment using a Snellen chart, objective and subjective refraction, slit-lamp biomicroscopy, intraocular pressure measurement using Goldmann applanation tonometry, and dilated fundus examination using indirect ophthalmoscopy.

Participants were eligible if they were 18–40 years of age, had no history of ocular disease, had a spherical equivalent refractive error within ±3.00 D, and had intraocular pressure within the normal physiological range (10–21 mm Hg). The refractive-error range was restricted to reduce the influence of more substantial refractive variation on anterior scleral morphology while retaining participants with low refractive errors commonly encountered in young adults. Because refractive status may still be associated with scleral thickness within this restricted range, spherical equivalent was included as a covariate in the adjusted linear mixed-effects model.

Participants were excluded if they had any ocular comorbidities such as narrow anterior chamber angles, elevated intraocular pressure (>21 mm Hg), refractive errors exceeding ±3.00 diopters spherical equivalent, astigmatism greater than 2.00 D, previous ocular surgery, glaucoma, ocular surface disease, dry eye syndrome, pinguecula, pterygium, retinal pathology, or a history of contact lens wear. Individuals unable to maintain stable fixation during imaging or whose scans were of insufficient quality were also excluded. Astigmatism greater than 2.00 D was excluded to limit substantial refractive variability that could potentially influence ocular biometric characteristics.

Anterior segment imaging was performed using a swept-source optical coherence tomography (SS-OCT) system (DRI OCT Triton, Topcon Corporation, Tokyo, Japan) equipped with an anterior segment module. A 6 mm line scan protocol within the anterior segment capture mode was utilized. OCT images were obtained from the superior, inferior, nasal, and temporal regions.

Each OCT scan was initiated at the region immediately adjacent to the scleral reflex and continued until the completion of a 6 mm segment in contact with the corneal surface. The duration of each scan was approximately 2 s. During imaging, participants were instructed to fixate on an external target positioned 1 m away to facilitate optimal exposure of the bulbar conjunctiva opposite to the direction of eye movement and reduce the effect of accommodation. Nasal eye movement exposed the temporal sclera, while upward eye movement revealed the inferior sclera, and vice versa.

Precise alignment of the line scan with the scleral reflex was performed for each participant to ensure consistency across measurements. Two horizontal scans (180°) were acquired for the nasal and temporal quadrants, whereas two vertical scans (90°) were obtained for the superior and inferior quadrants. For scans along the superior and inferior meridians, gentle lid retraction was performed when necessary to adequately expose the scleral surface.

All imaging procedures were conducted by a single trained examiner to maintain consistency and accuracy. Only high-quality images with a signal strength intensity (SSI) greater than 50 were included in the analysis. Real-time image quality assessment was performed, and scans were repeated when necessary to achieve optimal clarity. For each quadrant, two separate B-scan acquisitions were obtained consecutively during the same examination session, with an interval of approximately 1 min between acquisitions. During this interval, participants were instructed to close their eyes before the second scan was obtained. AST was measured independently on each B-scan by the same examiner at 0, 1, and 2 mm from the scleral spur. The examiner was not masked to the first measurement when performing the second measurement. The mean of the two readings was used for the primary statistical analysis, while the paired measurements were retained separately for assessment of short-term intra-observer test–retest repeatability and agreement across repeated image acquisitions. To minimize potential bias from inter-eye correlation, only right-eye data were included in the analysis, as measurements from the right and left eyes showed strong correlations across locations and quadrants (r = 0.81–0.87).

**Figure 1:**
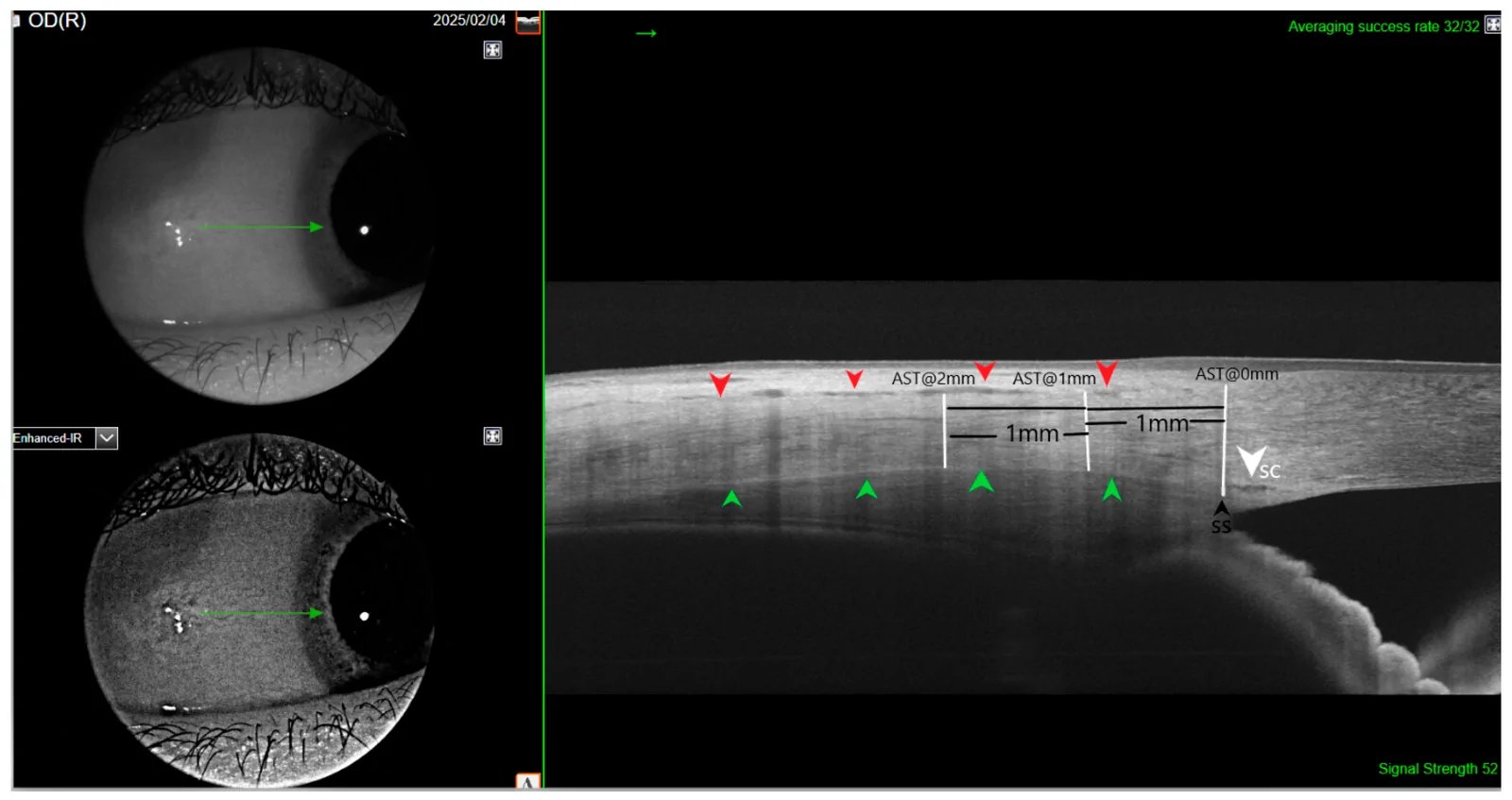
Measurement of anterior scleral thickness (AST) using swept-source optical coherence tomography (SS-OCT). Representative anterior segment B-scan of the right eye in the temporal quadrant showing AST measurements at the scleral spur (0 mm) and at 1 and 2 mm posterior to the scleral spur. The black arrow indicates the scleral spur (SS), which was used as the anatomical reference point, and the white arrow indicates Schlemm’s canal (SC). Red arrows indicate superficial conjunctival–episcleral vascular structures used as landmarks for identification of the outer scleral surface. Green arrows indicate the inner scleral boundary along the internal scleral contour. Vertical white bars indicate the 1 mm intervals used to define the measurement locations. The image shown is a representative scan obtained during preliminary protocol optimization and was not obtained from a participant included in the final study dataset.

Anterior scleral thickness (AST) was manually measured on the B-scan OCT images using IMAGEnet 6 software (version 1.22.1.14101, Topcon Corporation, Tokyo, Japan). The scleral boundaries were identified according to the characteristic OCT reflectivity pattern. The superficial conjunctival–episcleral vascular structures, indicated by the red arrows, were used as anatomical landmarks to identify the outer scleral surface rather than being considered part of the scleral boundary itself. The outer scleral boundary was defined at the transition between the superficial conjunctival–episcleral tissue and the more homogeneous hyper-reflective scleral stroma. At the 0 mm location, the inner scleral boundary was identified along the internal scleral contour at the scleral spur region, immediately posterior to the trabecular meshwork and Schlemm’s canal and adjacent to the anterior insertion of the ciliary muscle. At 1 and 2 mm posterior to the scleral spur, the inner scleral boundary was identified at the interface between the hyper-reflective scleral stroma and the underlying hypo-reflective ciliary body. These inner scleral boundaries are indicated by the green arrows in Figure 1. AST was measured manually as the perpendicular distance between the identified outer and inner scleral boundaries at the scleral spur (0 mm) and at 1 and 2 mm posterior to the scleral spur. The identification of scleral boundaries and measurement landmarks was based on previously described OCT-based anterior scleral thickness methodology [14].

The SS served as the reference plane, from which three consecutive measurements were taken at 1 mm intervals, as shown in the black text in Figure 1 (AST@0mm, AST@1mm, AST@2mm). AST measurements were obtained at 0 mm, 1 mm, and 2 mm from the scleral spur in nasal, temporal, superior, and inferior quadrants. All the measurements were taken between 2 pm and 4 pm to avoid diurnal variability.

### 2.1. Statistical Analysis

Statistical analyses were performed using IBM SPSS Statistics for Windows, version 27.0 (IBM Corp., Armonk, NY, USA). Continuous variables were summarized as mean ± standard deviation (SD), with confidence intervals where appropriate, and categorical variables as frequencies and percentages. AST was analyzed using a linear mixed-effects model to account for the 12 repeated quadrant-by-distance measurements obtained from each participant. Sex was included as a between-subject factor, while distance from the scleral spur (0, 1, and 2 mm) and quadrant (superior, inferior, nasal, and temporal) were included as within-subject factors. The model included the main effects of sex, distance, and quadrant and their two-way and three-way interactions. Age and spherical equivalent were included as covariates, and a participant-specific random intercept was included to account for clustering of repeated measurements within participants.

Three residual covariance structures were compared using an identical fixed-effects and random-effects specification: diagonal, unstructured, and a Kronecker product structure comprising an unstructured covariance across quadrants and a first-order autoregressive [AR(1)] covariance across measurement distances. Models were estimated using restricted maximum likelihood, and model fit was compared using the Akaike Information Criterion (AIC) and Bayesian Information Criterion (BIC), with lower values indicating better fit. The Kronecker UN × AR(1) structure provided the lowest AIC and BIC and was therefore retained for the final model. Denominator degrees of freedom were estimated using the Satterthwaite approximation. Model assumptions were evaluated using normal Q–Q plots of residuals and plots of residuals against predicted values.

Estimated marginal means were used to explore significant interactions. Because the sex × distance × quadrant interaction was significant, sex differences were evaluated separately at each of the 12 quadrant-by-distance locations. These 12 comparisons constituted a single family for Bonferroni correction. Adjusted *p* values were calculated, controlling the family-wise error rate at 0.05, and corresponding Bonferroni-adjusted 99.583% confidence intervals were reported. For quadrant comparisons, pairwise comparisons were performed separately within each sex-by-distance stratum. The six possible quadrant comparisons within each stratum constituted a separate family for Bonferroni adjustment.

Intra-observer repeatability of the manual AST measurements was evaluated separately at each measurement location using a two-way random-effects, absolute-agreement, single-measure intraclass correlation coefficient [ICC(A,1)] with 95% confidence intervals. The within-subject standard deviation was calculated from the standard deviation of the paired differences divided by √2, and the within-subject coefficient of variation (CoV) was calculated as the within-subject standard deviation divided by the overall mean and expressed as a percentage. The agreement between the two repeated measurements was further assessed using Bland–Altman analysis. Measurement bias was calculated as the mean difference between Reading 1 and Reading 2, and the 95% limits of agreement were calculated as the mean difference ± 1.96 standard deviations of the paired differences. The repeatability coefficient was calculated as 1.96 times the standard deviation of the paired differences. Statistical significance was defined as *p* < 0.05.

### 2.2. Use of Artificial Intelligence

Artificial intelligence (AI) tools (ChatGPT 5.0) were used only to improve the language, grammar, and readability of the manuscript. AI was not used for data collection, analysis, or interpretation. All AI-assisted content was reviewed and verified by the authors.

## 3. Results

**Figure 2:**
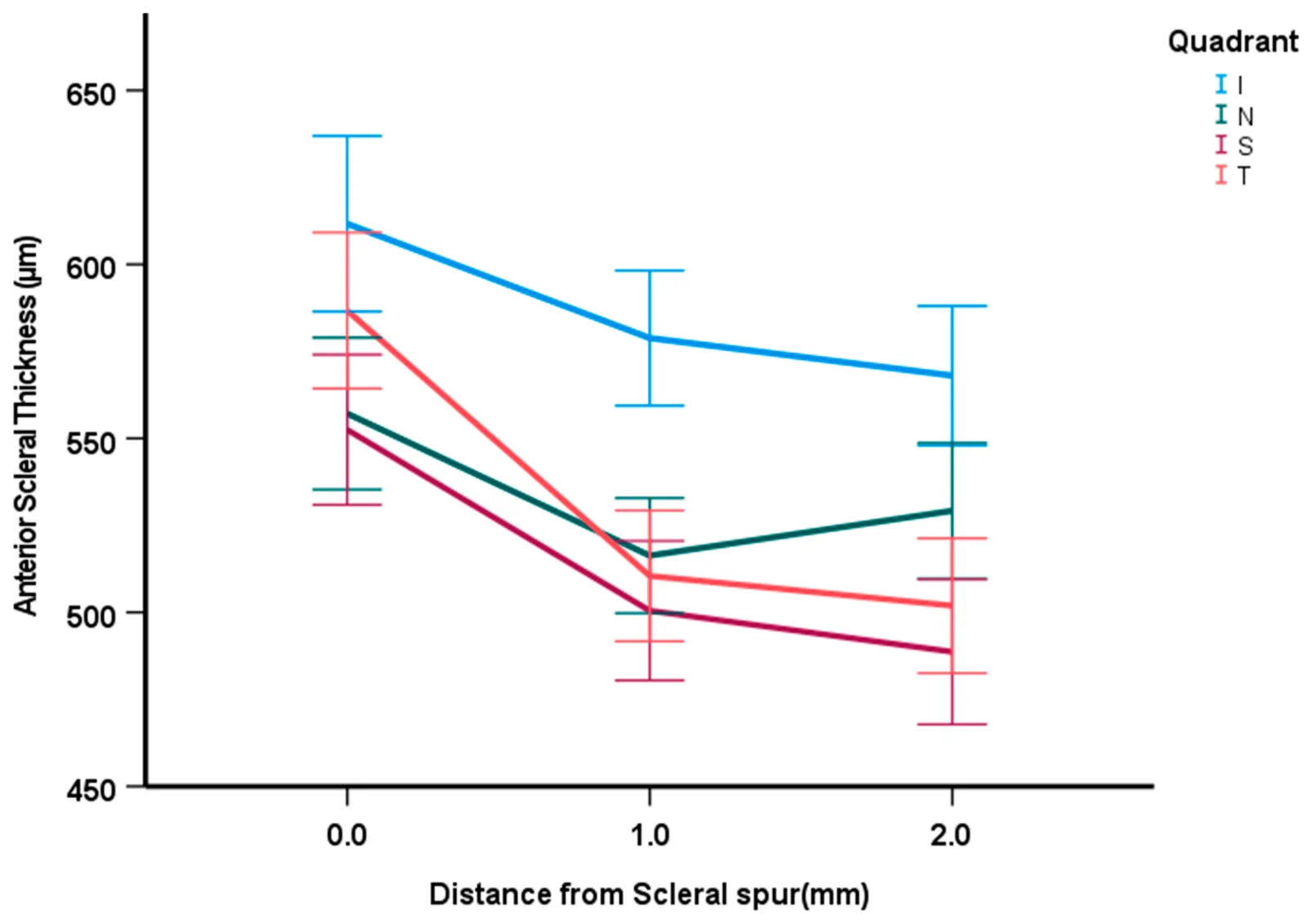
Anterior scleral thickness (AST) across measurement distances from the scleral spur according to anatomical quadrant. Mean AST values are shown for the superior, inferior, nasal, and temporal quadrants at 0, 1, and 2 mm from the scleral spur. The figure illustrates the differing pattern of AST change with distance across quadrants, consistent with the significant distance-by-quadrant interaction observed in the linear mixed-effects model. Note: I = inferior; N = nasal; S = superior; T = temporal. Error bars represent 95% confidence intervals.

The current study comprises 55 subjects with a mean age of 23.56 ± 4.61 years. Out of 55 study participants, 34 were males and 21 were females. The mean spherical equivalent of the right eye was −0.98 ± 0.87D and the mean intraocular pressure was 13.82 ± 1.78 mmHg (range, 10–19 mmHg). Anterior scleral thickness demonstrated a clear quadrant-dependent pattern across all measured distances from the scleral spur. The descriptive AST values for all quadrants and measurement points are presented in Table 1, and the corresponding graphical trends are shown in Figure 2.

The study included 55 eyes, with a mean spherical equivalent of −0.98 ± 0.87 D. At 0 mm from the scleral spur, anterior scleral thickness was highest in the inferior quadrant (611.71 ± 93.31 µm; 95% CI: 586.48–636.93), followed by the temporal quadrant (586.78 ± 82.96 µm; 95% CI: 564.35–609.20). The nasal quadrant measured 557.18 ± 80.76 µm with a 95% CI of 535.35–579.01, while the superior quadrant showed the lowest mean thickness at this location (552.53 ± 79.81 µm; 95% CI: 530.95−574.10) (Table 1; Figure 2).

At 1 mm from the scleral spur, the inferior quadrant again demonstrated the greatest thickness (578.85 ± 71.76 µm; 95% CI: 559.45–598.25). The nasal quadrant showed a mean thickness of 516.35 ± 61.18 µm with a 95% CI of 499.81–532.88, followed by the temporal quadrant (510.51 ± 69.56 µm; 95% CI: 491.70–529.31). The superior quadrant had the lowest mean value at this distance (500.51 ± 74.18 µm; 95% CI: 480.45–520.56) (Table 1; Figure 2).

At 2 mm from the scleral spur, the overall distribution remained consistent. The inferior quadrant continued to show the highest anterior scleral thickness (568.07 ± 74.10 µm; 95% CI: 548.04–588.10). This was followed by the nasal quadrant (529.22 ± 72.16 µm; 95% CI: 509.71–548.73) and the temporal quadrant (501.95 ± 71.71 µm; 95% CI: 482.56–521.33). The superior quadrant demonstrated the lowest mean thickness at 488.69 ± 77.16 µm, with a 95% CI of 467.83–509.55 (Table 1; Figure 2).

Overall, anterior scleral thickness decreased from the scleral spur towards 2 mm in the superior, inferior, and temporal quadrants. In contrast, the nasal quadrant showed a non-linear pattern, decreasing from 557.18 µm at the scleral spur to 516.35 µm at 1 mm, followed by a modest increase to 529.22 µm at 2 mm. The inferior quadrant remained the thickest region at all three measurement locations, whereas the superior quadrant showed the lowest values, particularly at 1 mm and 2 mm from the scleral spur. These findings indicate a non-uniform distribution of anterior scleral thickness, with relative thickening inferiorly and thinning superiorly across the anterior sclera (Table 1; Figure 2).

### 3.1. Linear Mixed-Effects Analysis of AST

Three residual covariance structures were compared using the same fixed-effects specification and participant-specific random intercept. The Kronecker UN × AR(1) model showed the lowest AIC and BIC (AIC = 6970.534; BIC = 7023.958) compared with the diagonal (AIC = 7097.033; BIC = 7154.910) and unstructured models (AIC = 7102.073; BIC = 7453.785) and was therefore retained for the final analysis. The estimated AR(1) correlation across measurement distances was 0.570, and the participant-specific random-intercept variance was 579.02 µm^2^ (SE = 635.64). Residual Q–Q plots showed approximate normality, supported by a nonsignificant Shapiro–Wilk test (*p* = 0.091), while inspection against predicted values showed no pronounced systematic pattern or marked funneling.

In the final model, significant effects were observed for sex [F(1, 46.935) = 4.421, *p* = 0.041], distance from the scleral spur [F(2, 102.196) = 39.098, *p* < 0.001], and quadrant [F(3, 93.851) = 31.503, *p* < 0.001]. Significant sex-by-distance [F(2, 102.196) = 14.508, *p* < 0.001], sex-by-quadrant [F(3, 93.851) = 2.963, *p* = 0.036], distance-by-quadrant [F(6, 133.231) = 7.016, *p* < 0.001], and sex-by-distance-by-quadrant interactions [F(6, 133.231) = 2.957, *p* = 0.010] were observed. Neither age [F(1, 44.815) = 0.074, *p* = 0.787] nor spherical equivalent [F(1, 44.815) = 0.130, *p* = 0.720] showed a significant independent association with AST. Given the significant sex-by-distance-by-quadrant interaction, interpretation of sex-related differences focused on location-specific comparisons rather than on the overall sex main effect or the separate two-way interactions (Table 2).

Bonferroni-adjusted pairwise comparisons showed that quadrant differences varied according to both sex and measurement distance. In females, at 0 mm, the inferior and nasal quadrants were significantly thicker than the superior quadrant, while the temporal quadrant was also significantly thicker than the superior quadrant. At 1 mm, the inferior quadrant was significantly thicker than the nasal, superior, and temporal quadrants. At 2 mm, the inferior quadrant was significantly thicker than the superior and temporal quadrants, while the nasal quadrant was significantly thicker than both the superior and temporal quadrants. In males, at 0 mm, the inferior quadrant was significantly thicker than the nasal and superior quadrants, while the temporal quadrant was significantly thicker than the nasal quadrant. At both 1 mm and 2 mm, the inferior quadrant was significantly thicker than the nasal, superior, and temporal quadrants. Detailed sex- and distance-specific quadrant comparisons are presented in Supplementary Table S1.

Because the sex-by-distance-by-quadrant interaction was significant, sex differences were examined separately at each of the 12 measurement locations. Following Bonferroni adjustment across these 12 comparisons, males demonstrated significantly greater adjusted AST in the superior and temporal quadrants at both 1 mm and 2 mm from the scleral spur. No significant sex differences were observed at 0 mm or in the inferior or nasal quadrants at 1 mm and 2 mm (Table 3). Sex-stratified descriptive AST values (mean ± SD) for all 12 measurement locations are presented in Supplementary Table S2.

### 3.2. Intra-Observer Repeatability and Agreement

Short-term within-session intra-observer test–retest repeatability across repeated B-scan acquisitions was generally good across the 12 AST measurement locations. ICC(A,1) values ranged from 0.872 to 0.939, and the within-subject CoV ranged from 3.39% to 5.70%. The highest ICC was observed in the temporal quadrant at 1 mm (ICC = 0.939; 95% CI, 0.908–0.961), whereas the lowest was observed in the superior quadrant at the scleral spur (ICC = 0.872; 95% CI, 0.799–0.913). Bland–Altman analysis showed small mean biases between repeated measurements, ranging from −7.27 to 7.96 µm. The 95% limits of agreement varied according to measurement location, with repeatability coefficients ranging from 48.26 to 86.91 µm (Table 4).

## 4. Discussion

This study provides baseline data on anterior scleral thickness (AST) measured using swept-source optical coherence tomography in a young, healthy Saudi cohort. Although scleral thickness has been evaluated previously in adult populations, to the best of our knowledge, this is among the first studies to specifically describe AST patterns in Saudi individuals. These findings may therefore contribute to a better understanding of baseline anterior scleral thickness distribution in young Saudi adults.

In the present study, AST showed significant variation according to both measurement distance and anatomical quadrant. The mixed-effects model demonstrated significant main effects of distance and quadrant, together with a significant distance-by-quadrant interaction, indicating that the pattern of AST change with increasing distance from the scleral spur was not identical across quadrants. The inferior quadrant generally demonstrated the greatest thickness, whereas the superior quadrant showed lower values, particularly at 1 mm and 2 mm. AST generally decreased with increasing distance from the scleral spur in the superior, inferior, and temporal quadrants, while the nasal quadrant showed a reduction from 0 to 1 mm followed by a modest increase at 2 mm. This regional pattern is broadly consistent with previous in vivo [11,12,13,14,15,16,17,18] and ex vivo [19,20,21] studies that used different imaging and measurement techniques.

The quadrant-based distribution observed in this study is also consistent with earlier reports. In the pooled descriptive data, AST was greatest along the inferior meridian and lowest along the superior meridian at all three measurement distances; however, sex-specific pairwise comparisons demonstrated that the pattern of regional differences varied according to sex and distance from the scleral spur. Dhakal et al. [11] reported a similar pattern, with the anterior sclera being thinnest superiorly and thickest inferiorly, and this trend remained consistent up to 5 mm from the scleral spur. Biometry-based investigations [14] also demonstrated inferior scleral thickening relative to the superior quadrant, with the nasal sclera generally thicker than the temporal sclera. Similarly, Buckhurst et al. [16], using anterior segment optical coherence tomography, found AST to be greatest inferiorly and thinnest in the superior-nasal region, with intermediate values along the nasal and temporal meridians. The higher absolute values reported in their study may be explained by the inclusion of episcleral tissue, likely due to the lower axial resolution of the imaging system used. Overall, these findings support a broadly similar regional pattern across populations, although the magnitude of quadrant differences may vary according to population characteristics and imaging methodology.

From a clinical perspective, the present findings should primarily be considered anatomical baseline information rather than diagnostic thresholds. Nevertheless, characterization of regional AST may provide useful context for future investigations involving disorders or procedures that affect the anterior sclera. In inflammatory conditions such as scleritis and episcleritis, comparison with healthy regional thickness patterns may assist future studies in determining whether localized thickening differs from physiological quadrant-related variation. In myopia research, anterior scleral measurements may complement studies of posterior scleral remodeling and axial elongation by providing information about whether structural changes extend to the anterior ocular wall. Similarly, studies of glaucoma may investigate whether variations in anterior scleral morphology are associated with ocular biomechanics or structures related to aqueous outflow. For scleral and semi-scleral contact lenses, AST should not be considered a direct measure of scleral shape; however, regional thickness information may complement elevation and contour measurements when investigating the interaction between the lens and underlying ocular tissues. Finally, baseline anatomical information may also be useful for future studies of procedures involving the anterior sclera. These potential applications require dedicated clinical studies, as the present cross-sectional study did not evaluate diagnostic performance, treatment outcomes, contact lens fitting, or surgical safety [7,9,11,22,23,24,25,26,27].

Sex-related differences in AST were location-dependent rather than uniform across the anterior sclera. The final mixed-effects model demonstrated a significant sex-by-distance-by-quadrant interaction, indicating that the magnitude of the sex difference depended jointly on anatomical quadrant and distance from the scleral spur. Following Bonferroni correction across the 12 location-specific comparisons, males showed significantly greater adjusted AST in the superior and temporal quadrants at both 1 mm and 2 mm from the scleral spur. No significant sex differences were observed at 0 mm or in the inferior or nasal quadrants at 1 mm and 2 mm. These findings indicate that sex-related variation in AST is region-specific and becomes more apparent at selected locations posterior to the scleral spur. Measurements obtained at the scleral spur should be interpreted with additional caution because the local anatomy is more complex than at posterior measurement locations, and identification of the inner scleral boundary may be more sensitive to landmark placement.

The observed location-dependent sex differences are broadly consistent with previous studies reporting greater scleral thickness in males than females in selected anterior scleral regions [16,18,22]. The mechanisms underlying these differences remain uncertain and may relate to sex-associated variation in ocular dimensions, collagen organization, extracellular matrix composition, or hormonal influences on connective tissue remodeling. Importantly, the present findings indicate that sex-related differences should not be interpreted as a uniform increase in AST across the entire anterior sclera but rather as a location-specific pattern determined by the combined effects of measurement distance and quadrant.

These findings highlight the importance of considering anatomical quadrant, measurement distance, and sex jointly when interpreting AST measurements. Interpretation based on pooled AST values may overlook physiological variation, particularly where sex-related differences are more evident. The present anatomical data may provide useful context for future studies evaluating AST in conditions such as myopia, glaucoma, and inflammatory scleral disease.

Age was not independently associated with AST in the adjusted mixed-effects model. Similarly, spherical equivalent did not show a significant independent association with AST. These findings should be interpreted cautiously because the study population had a relatively narrow age range and restricted refractive-error distribution. Therefore, the absence of significant associations in the present sample should not be interpreted as evidence that age or refractive status has no influence on AST in a broader population.

An additional strength of the present study was the quantitative assessment of intra-observer repeatability of the manual AST measurements. ICC values indicated generally good repeatability across all measurement locations, while the relatively low within-subject CoV values supported the consistency of the repeated measurements. Bland–Altman analysis demonstrated small systematic biases between the two readings. Nevertheless, the width of the limits of agreement varied among measurement locations, indicating that absolute measurement variability was not uniform throughout the anterior sclera. This location-dependent variability should therefore be considered when interpreting relatively small differences in AST. The repeatability coefficients ranged from 48.26 to 86.91 µm and were therefore of a similar magnitude to several of the observed group-level AST differences. Although this does not invalidate the group-level comparisons obtained from the mixed-effects model, it limits interpretation at the individual level. Relatively small differences in AST measured in an individual eye may fall within the expected short-term test–retest measurement variability, and differences smaller than the location-specific repeatability coefficient should therefore not be considered definite biological change or used as individual diagnostic thresholds.

This study has several limitations. First, the relatively narrow and young age distribution limits interpretation of age-related associations with AST. Second, AST measurements were limited to 2 mm from the reference point; therefore, mid-peripheral and peripheral scleral thickness were not evaluated. Third, axial length was not measured; therefore, residual confounding by ocular size cannot be excluded, particularly when interpreting the observed sex-related differences in AST. The unequal sex distribution may also have reduced the precision of sex-specific estimates, particularly among female participants. In addition, the observed variability in AST was greater than that assumed in the original sample-size calculation (observed SDs approximately 61–93 µm versus an assumed SD of 46 µm). Consequently, the achieved 95% confidence-interval precision was approximately ±16 to ±25 µm for the overall estimates, rather than the prespecified ±15 µm, with lower precision in the smaller female subgroup. The effect of intraocular pressure was also not specifically examined. However, IOP values were within the normal range in this cohort (mean 13.82 ± 1.78 mmHg; range, 10–19 mmHg). The restricted age range and predominance of young adults limit the generalizability of the age-related findings to middle-aged and older populations. Finally, although participants originated from different regions of Saudi Arabia, recruitment was conducted through a single university-associated center; therefore, the sample may not be fully representative of the broader Saudi population or populations from other geographical regions. Although intra-observer repeatability was generally good, AST measurements were performed manually by a single examiner, and the location-dependent limits of agreement indicate that some measurement variability remains. In addition, the examiner was not masked to the first measurement when performing the second reading, which may have introduced some measurement bias.

Several directions for future research arise from the present findings. First, multicenter studies involving larger and more geographically diverse Saudi populations would be useful to determine the reproducibility of the observed regional AST patterns and to provide more precise population-based estimates. Broader age groups should also be included to characterize age-related changes across adulthood, as the present cohort predominantly represents young adults. Second, future studies should incorporate axial length and additional ocular biometric parameters so that the independent effects of ocular size, refractive status, and sex on AST can be examined more comprehensively. Recruitment of balanced numbers of male and female participants and inclusion of defined refractive groups would further improve these analyses. Third, measurements extending beyond 2 mm from the scleral spur could determine whether the regional pattern observed in the present study continues into the mid-peripheral sclera. Longitudinal studies may also clarify whether AST changes over time or in association with refractive progression. From a methodological perspective, inter-observer reproducibility and automated or semi-automated scleral boundary segmentation should be investigated to reduce operator dependence. Finally, comparison of healthy participants with individuals with myopia, glaucoma, and inflammatory scleral disorders may help establish whether disease-related AST changes exceed the physiological regional variation observed in healthy eyes.

In conclusion, AST in young Saudi adults varies according to anatomical quadrant and distance from the scleral spur, with sex-related differences showing location-specific patterns. The inferior sclera generally demonstrated greater thickness, while the superior sclera showed lower values. Adjusted mixed-effects analysis demonstrated a significant sex-by-distance-by-quadrant interaction, with males showing greater AST specifically in the superior and temporal quadrants at both 1 mm and 2 mm from the scleral spur after correction for multiple comparisons. These findings emphasize the importance of considering the combined effects of anatomical location, measurement distance, and sex when interpreting anterior scleral thickness measurements.

## Figures and Tables

**Table 1:** Anterior scleral thickness according to quadrant and distance from the scleral spur.

Distance from Scleral Spur	Quadrant	Mean ± SD (µm)	95% CI of Mean (µm)
0 mm	Superior	552.53 ± 79.81	530.95–574.11
	Inferior	611.71 ± 93.31	586.48–636.94
	Temporal	586.78 ± 82.96	564.35–609.21
	Nasal	557.18 ± 80.76	535.35–579.01
1 mm	Superior	500.51 ± 74.18	480.46–520.56
	Inferior	578.85 ± 71.76	559.45–598.25
	Temporal	510.51 ± 69.56	491.71–529.31
	Nasal	516.35 ± 61.18	499.81–532.89
2 mm	Superior	488.69 ± 77.16	467.83–509.55
	Inferior	568.07 ± 74.10	548.04–588.10
	Temporal	501.95 ± 71.71	482.56–521.34
	Nasal	529.22 ± 72.16	509.71–548.73

Note: Values are presented as mean ± standard deviation (SD) with the corresponding 95% confidence interval (CI) of the mean. AST, anterior scleral thickness; n = 55.

**Table 2:** Linear mixed-effects model of factors associated with anterior scleral thickness.

Fixed Effect	Numerator df	Denominator df	F	*p* Value
Sex	1	46.935	4.421	**0.041**
Distance from scleral spur	2	102.196	39.098	**<0.001**
Quadrant	3	93.851	31.503	**<0.001**
Age	1	44.815	0.074	0.787
Spherical equivalent	1	44.815	0.130	0.720
Sex × Distance	2	102.196	14.508	**<0.001**
Sex × Quadrant	3	93.851	2.963	**0.036**
Distance × Quadrant	6	133.231	7.016	**<0.001**
Sex × Distance × Quadrant	6	133.231	2.957	**0.010**

**Note:** AST was the dependent variable. The model included a participant-specific random intercept and a Kronecker product residual covariance structure comprising an unstructured covariance across quadrants and a first-order autoregressive [AR(1)] covariance across measurement distances. Age and spherical equivalent were included as covariates. Restricted maximum likelihood estimation and the Satterthwaite approximation for denominator degrees of freedom were used. Bold *p* values indicate statistical significance at *p* < 0.05.

**Table 3:** Adjusted location-specific sex differences in anterior scleral thickness.

Distance from Scleral Spur	Quadrant	Male–Female Difference (µm)	99.583% CI (µm)	Bonferroni-Adjusted *p* Value
0 mm	Inferior	−18.92	−82.40–4.56	1.000
0 mm	Nasal	−55.36	−119.09–8.37	0.149
0 mm	Superior	28.78	−27.91–85.47	1.000
0 mm	Temporal	−25.96	−86.16–34.23	1.000
1 mm	Inferior	52.80	−10.68–116.28	0.200
1 mm	Nasal	41.89	−21.84–105.62	0.681
1 mm	Superior	**68.65**	**11.96–125.34**	**0.007**
1 mm	Temporal	**61.10**	**0.91–121.30**	**0.044**
2 mm	Inferior	36.13	−27.35–99.61	1.000
2 mm	Nasal	31.13	−32.60–94.86	1.000
2 mm	Superior	**64.17**	**7.48–120.86**	**0.015**
2 mm	Temporal	**82.69**	**22.49–142.88**	**0.001**

**Note:** Values represent differences in estimated marginal means (male minus female) from the final linear mixed-effects model adjusted for age and spherical equivalent. Twelve sex comparisons (3 distances × 4 quadrants) were treated as a single family for Bonferroni correction. Adjusted *p* values and corresponding 99.583% simultaneous confidence intervals are reported. Positive differences indicate greater anterior scleral thickness in males. Bold values indicate statistically significant comparisons after Bonferroni adjustment.

**Table 4:** Intra-observer repeatability and agreement of anterior scleral thickness measurements.

Distance from Scleral Spur	Quadrant	ICC (A,1)	95% CI	CoV (%)	Bias R1−R2 (µm)	Lower 95% LoA (µm)	Upper 95% LoA (µm)	Repeatability Coefficient (µm)
0 mm	S	0.872	0.799–0.913	5.40	0.22	−82.42	82.85	82.64
0 mm	I	0.892	0.841–0.927	5.13	7.96	−78.95	94.87	86.91
0 mm	T	0.936	0.895–0.959	3.67	1.78	−57.85	61.42	59.63
0 mm	N	0.914	0.863–0.942	4.32	−5.82	−72.54	60.90	66.72
1 mm	S	0.881	0.800–0.928	5.26	5.89	−67.07	78.86	72.96
1 mm	I	0.900	0.845–0.932	4.04	3.67	−61.16	68.51	64.83
1 mm	T	0.939	0.908–0.961	3.41	−3.56	−51.83	44.70	48.26
1 mm	N	0.921	0.870–0.949	3.39	3.71	−44.78	52.19	48.48
2 mm	S	0.876	0.782- 0.925	5.70	−7.27	−84.44	69.90	77.17
2 mm	I	0.905	0.846–0.939	4.06	7.20	−56.74	71.14	63.94
2 mm	T	0.931	0.878–0.961	3.84	1.67	−51.72	55.07	53.40
2 mm	N	0.908	0.838–0.943	4.28	0.15	−62.58	62.87	62.72

Note: ICC, intraclass correlation coefficient; CI, confidence interval; CoV, coefficient of variation; LoA, limits of agreement. ICC(A,1) denotes a two-way random-effects, absolute-agreement, single-measure ICC. CoV was calculated as the within-subject standard deviation divided by the overall mean × 100. Bland–Altman bias was calculated as Reading 1–Reading 2; the 95% LoA were calculated as bias ± 1.96 × SD of the paired differences. The repeatability coefficient was calculated as 1.96 × SD of the paired differences.

## Data Availability

The data supporting the findings of this study are available from the corresponding author upon request.

## Supplementary Materials

The following supporting information can be downloaded at: https://www.mdpi.com/article/10.3390/jcm15207847/s1, Table S1. Bonferroni-adjusted pairwise comparisons of anterior scleral thickness between quadrants according to sex and distance from the scleral spur; Table S2. Sex-specific anterior scleral thickness at different distances from the scleral spur.
