# Supplementary material for: Anterior Scleral Thickness in Young Saudi Adults Measured Using Swept-Source Optical Coherence Tomography: Sex- and Quadrant-Specific Variations
Source: J Clin Med. 2026 Oct 12;15(20):7847. doi: 10.3390/jcm15207847 (PMC13705192; doi:10.3390/jcm15207847)
Supplement: Supplementary file 1 [file jcm-15-07847-s001.zip › jcm-4558449-supplementary.pdf]

Table S1. Bonferroni-adjusted pairwise comparisons of anterior scleral thickness between quadrants at each measurement distance

Distance from scleral spur	Quadrant comparison	Mean difference (I–J), µm	SE, µm	df	95% CI, µm	Adjusted p value
0 mm	Inferior − Nasal	50.221	15.350	93.130	8.841 to 91.600	0.009
0 mm	Inferior − Superior	64.819	14.460	92.381	25.831 to 103.806	<0.001
0 mm	Inferior − Temporal	24.095	15.151	93.169	-16.749 to 64.938	0.691
0 mm	Nasal − Superior	14.598	14.108	92.368	-23.441 to 52.638	1.000
0 mm	Nasal − Temporal	-26.126	14.816	93.222	-66.065 to 13.813	0.487
0 mm	Superior − Temporal	-40.724	13.892	92.063	-78.183 to -3.265	0.026
1 mm	Inferior − Nasal	61.219	8.089	80.918	39.341 to 83.097	<0.001
1 mm	Inferior − Superior	80.218	7.858	74.403	58.919 to 101.518	<0.001
1 mm	Inferior − Temporal	69.326	7.901	85.365	47.984 to 90.668	<0.001
1 mm	Nasal − Superior	18.999	8.766	90.661	-4.646 to 42.645	0.197
1 mm	Nasal − Temporal	8.107	8.805	82.964	-15.693 to 31.907	1.000
1 mm	Superior − Temporal	-10.892	8.593	83.840	-34.113 to 12.329	1.000
2 mm	Inferior − Nasal	38.263	10.024	81.452	11.157 to 65.369	0.002
2 mm	Inferior − Superior	82.695	9.333	82.457	57.466 to 107.925	<0.001
2 mm	Inferior − Temporal	71.629	8.670	87.905	48.225 to 95.032	<0.001
2 mm	Nasal − Superior	44.432	10.940	92.645	14.938 to 73.926	0.001
2 mm	Nasal − Temporal	33.366	10.380	84.630	5.321 to 61.410	0.011
2 mm	Superior − Temporal	-11.067	9.715	88.974	-37.282 to 15.149	1.000

Note: Values are based on estimated marginal means from the linear mixed-effects model. For each comparison, the mean difference is calculated as the first-listed quadrant minus the second-listed quadrant. P values and 95% confidence intervals are Bonferroni-adjusted for multiple comparisons. A negative mean difference indicates lower AST in the first-listed quadrant. AST, anterior scleral thickness; CI, confidence interval; SE, standard error.

Table S2. Sex-specific anterior scleral thickness at different distances from the scleral spur

Distance from SS	Quadrant	Female, Mean ± SD (μm)	Male, Mean ± SD (μm)
0 mm	Superior	533.67 ± 74.10	564.18 ± 82.04
	Inferior	622.33 ± 96.90	605.15 ± 91.87
	Temporal	601.76 ± 94.83	577.53 ± 74.71
	Nasal	590.33 ± 103.19	536.71 ± 55.57
1 mm	Superior	457.00 ± 53.82	527.38 ± 72.80
	Inferior	545.14 ± 52.00	599.68 ± 74.96
	Temporal	471.67 ± 54.55	534.50 ± 67.53
	Nasal	489.38 ± 52.01	533.00 ± 61.13
2 mm	Superior	447.95 ± 52.61	513.85 ± 79.72
	Inferior	544.67 ± 65.77	582.53 ± 76.15
	Temporal	449.76 ± 59.48	534.18 ± 58.88
	Nasal	508.90 ± 57.12	541.76 ± 78.22

Values are presented as mean ± standard deviation (SD). Female: n = 21; male: n = 34. AST, anterior scleral thickness ; SS, scleral spur.
